# Painful neuromas exhibit axonal phenotypic shift via nociceptive and mechanosensitive marker up-regulation

**DOI:** 10.1093/pnasnexus/pgag257

**Published:** 2026-08-11

**Authors:** Vlad Tereshenko, Floris V Raasveld, Charles D Hwang, Madison R Hussey, Benjamin Johnston, Ian L Valerio, William G Austen, Brian J Wainger, William Renthal, Kyle R Eberlin

**Affiliations:** Division of Plastic and Reconstructive Surgery, Massachusetts General Hospital, Harvard Medical School, Boston, MA 02114, USA; Department of Plastic, Reconstructive and Aesthetic Surgery, Vienna General Hospital, Medical University of Vienna, Vienna 1090, Austria; Division of Plastic and Reconstructive Surgery, Massachusetts General Hospital, Harvard Medical School, Boston, MA 02114, USA; Division of Plastic and Reconstructive Surgery, Massachusetts General Hospital, Harvard Medical School, Boston, MA 02114, USA; Division of Plastic and Reconstructive Surgery, Massachusetts General Hospital, Harvard Medical School, Boston, MA 02114, USA; Department of Plastic, Reconstructive and Aesthetic Surgery, Vienna General Hospital, Medical University of Vienna, Vienna 1090, Austria; Department of Neurosurgery, Mass General Brigham, Harvard Medical School, Boston, MA 02114, USA; Division of Plastic and Reconstructive Surgery, Massachusetts General Hospital, Harvard Medical School, Boston, MA 02114, USA; Division of Plastic and Reconstructive Surgery, Massachusetts General Hospital, Harvard Medical School, Boston, MA 02114, USA; Departments of Anesthesia, Critical Care and Pain Medicine and Neurology, Massachusetts General Hospital, Harvard Medical School, Boston, MA 02114, USA; Department of Neurology, Brigham and Women's Hospital and Harvard Medical School, Boston, MA 02115, USA; Division of Plastic and Reconstructive Surgery, Massachusetts General Hospital, Harvard Medical School, Boston, MA 02114, USA

**Keywords:** neuroma, peripheral nerve, Piezo2, nerve surgery, nociception

## Abstract

Neuropathic pain encompasses a spectrum of conditions that significantly impact quality of life. In case of nerve injuries, when regenerating axons fail to reach their target organs, they can form painful neuromas, yet the mechanisms underlying their formation remain unclear. In this exploratory study, we analyzed the neural components of painful neuromas by examining the expression patterns of key nociceptor markers. Neural components within neuromas exhibited up-regulated growth-associated protein 43, indicating their regenerative potential. Calcitonin gene-related peptide was expressed in 84% of axons, versus 3% in healthy nerves, suggesting a phenotypic shift toward a nociceptive profile. We also observed increased expression of the mechanosensitive channel Piezo2 in 78% of axons and up-regulation of the low-threshold sodium channel Nav1.3, consistent with heightened mechanosensitivity and hyperexcitability. These observations identify candidate molecular alterations in painful neuromas and may provide a guide for future development of targeted therapies for neuroma-related pain.

Significance statement Painful neuromas are a major cause of chronic neuropathic pain, yet the molecular changes that make injured nerves hyperactive and painful have remained unclear. By analyzing human neuroma tissue, we show that axons within neuroma show a distinct program of growth, mechanosensitivity, and excitability, including up-regulation of growth-associated protein 43, calcitonin gene-related peptide, Piezo2, and Nav1.3. These findings demonstrate that axons of painful neuromas are reprogrammed into nociceptive states, offering a glimpse into the understanding of neuroma-associated pain and identifying molecular targets for more precise, mechanism-based therapies.

## Introduction

A remarkable evolutionary feature of the peripheral nervous system is its ability to support axonal regeneration after nerve injury (1, 2). In various species, this capacity serves as a crucial mechanism for functional recovery, reaching its pinnacle in tetrapods, such as the axolotl, which can regenerate entire limbs following amputation (3). While the mechanisms underlying such advanced regeneration remain under investigation, they hold promise for potential applications in restoring extremity function in humans (4). Despite not achieving full-limb regeneration, humans retain a significant regenerative capacity in peripheral nerve repair, allowing injured axons to regrow after trauma (5). This ability serves as a valuable biological tool in clinical practice, enabling surgical interventions to restore sensory and motor function (6–8). However, this regenerative potential also has a major drawback: when regenerating axons fail to reach their target organs, they can form painful neuromas (9). These neuromas often arise in untreated nerve injuries or following amputations, leading to debilitating neuropathic pain.

While the association between failed axonal target reinnervation and neuroma formation is well recognized, the underlying mechanisms remain elusive (9). Nevertheless, emerging surgical techniques, such as targeted muscle reinnervation (TMR) and regenerative peripheral nerve interfaces (RPNIs), have shown promise in preventing symptomatic neuroma formation by guiding regenerating axons toward muscle targets (10–15). Clinical data suggest that reestablishing axonal continuity reduces pain, yet the molecular mechanisms underlying this therapeutic benefit remain unclear (16).

The neurobiology of painful neuromas remains an area of active investigation. Although neuroma-associated pain has been observed in various peripheral nerves, the molecular pathways driving this process are not fully understood (9, 17, 18). Recent studies suggest that neuroma formation involves the up-regulation and down-regulation of ion channels, neurotransmitters, and inflammatory peptides, leading to transcriptomic reprogramming of injured axons (9, 19, 20). However, key questions remain, including whether nociceptive remodeling is initiated peripherally within the neuroma or centrally at the dorsal root ganglia. Furthermore, no definitive molecular markers have been identified that distinguish neuromas from healthy peripheral nerves.

**Figure 1:**
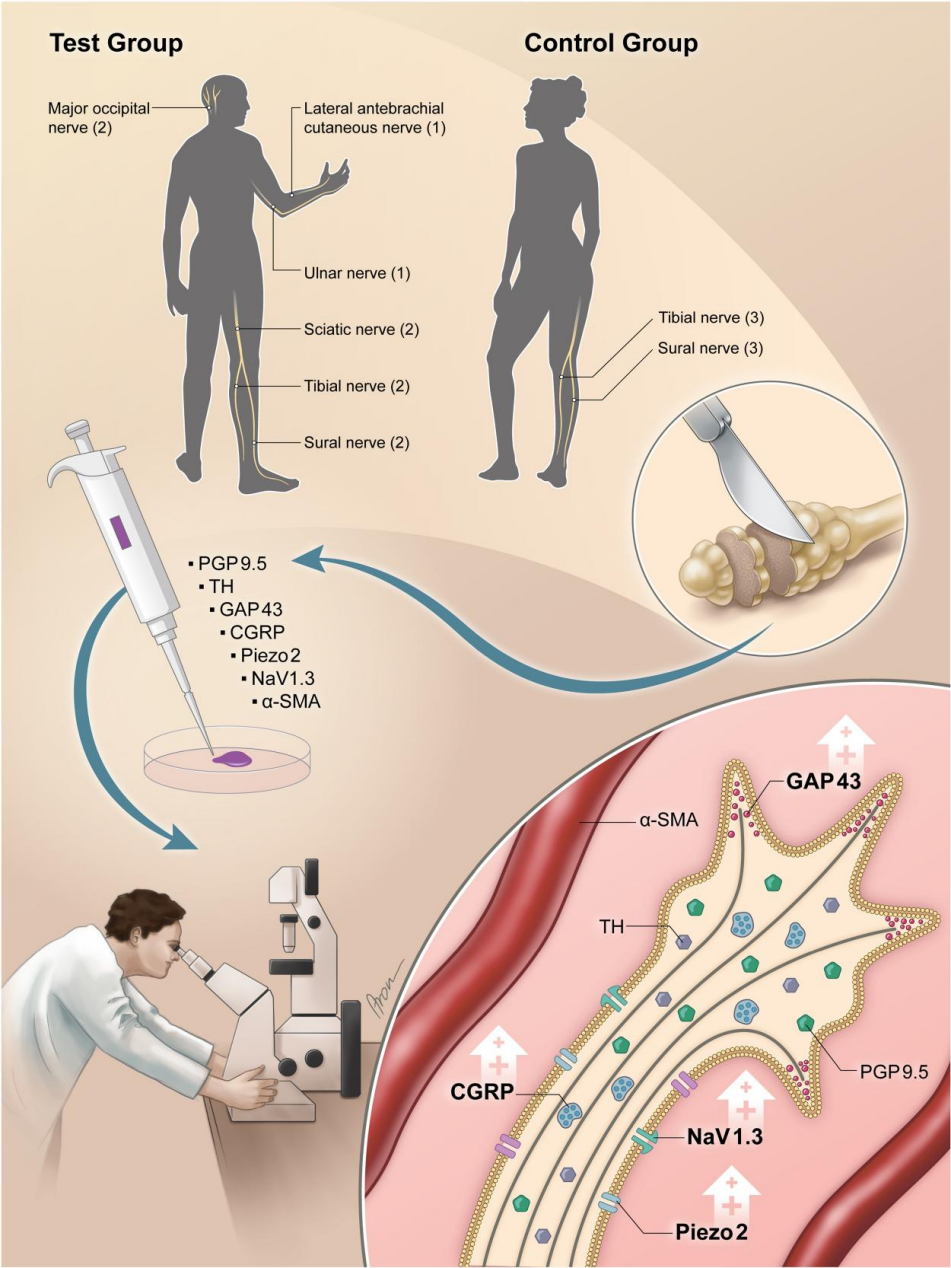
Study design. The unique molecular characteristics of painful neuromas were analyzed using specific molecular markers via immunofluorescent confocal microscopy. Painful neuromas were harvested from patients undergoing active nerve management and compared with healthy nerves obtained from patients undergoing lower extremity amputations. Both neuroma and healthy nerve specimens were processed and sectioned at 50 µm thickness for immunofluorescent staining using the molecular markers indicated above. Confocal microscopy revealed up-regulation in painful neuromas of GAP43 (associated with axonal regeneration), CGRP (involved in nociceptive signaling), Nav1.3 (linked to neuronal hyperexcitability), and Piezo2 (associated with mechanosensitive pain), compared with healthy nerves.

This study aims to show distinguished molecular architecture between painful neuromas and healthy nerves using immunofluorescence imaging (Fig. 1). By identifying key nociceptor drivers, we hope to advance the understanding of neuropathic pain in neuromas and pave the way for targeted therapeutic interventions to improve clinical management and patient outcomes.

## Results

### Disorganized axonal architecture with increased regenerative capacity in painful neuromas

**Figure 2:**
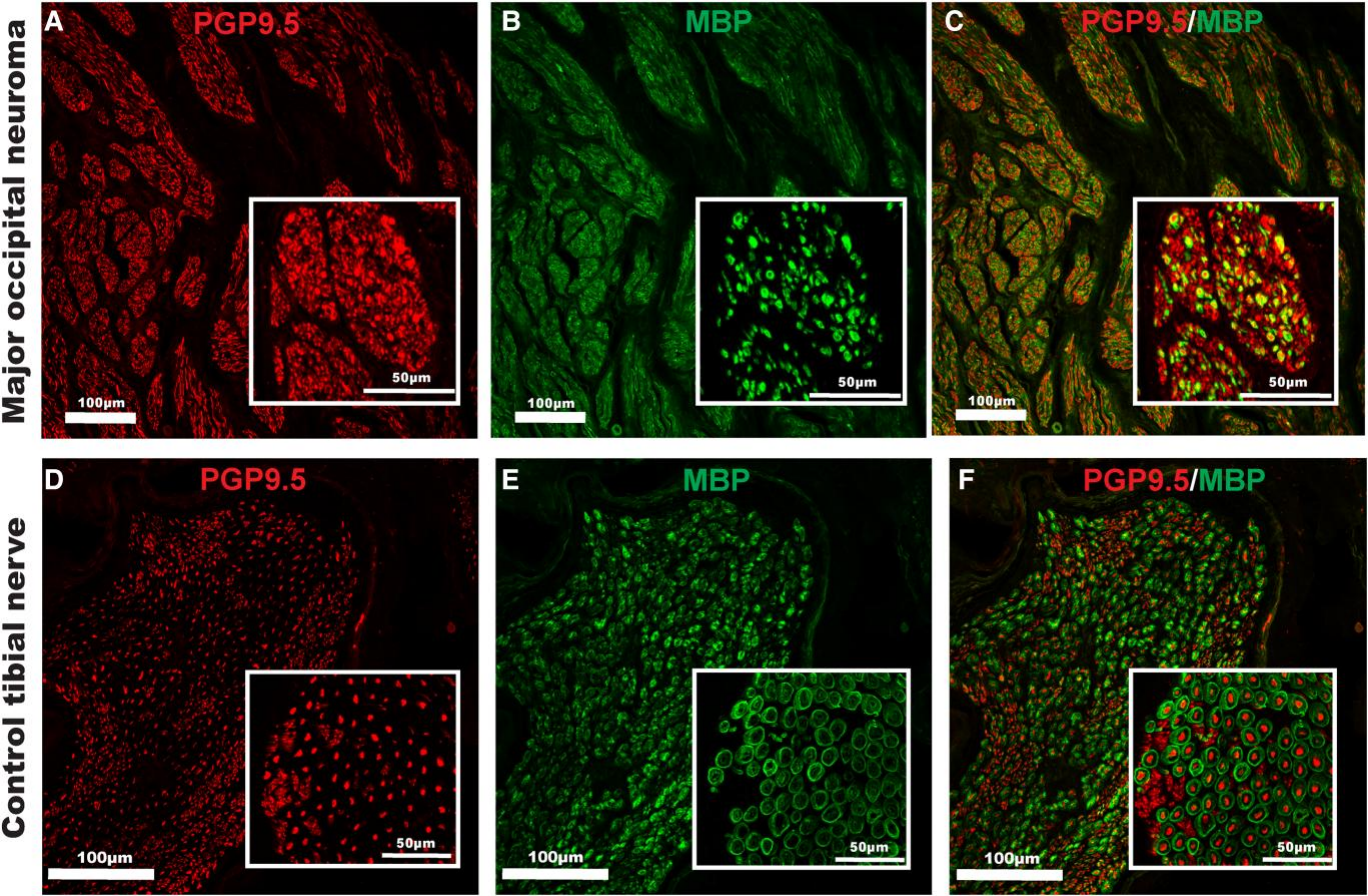
Axonal organization in painful neuromas compared with healthy nerves. Representative images of a major occipital neuroma (A–C) reveal a disorganized axonal architecture, as indicated by PGP9.5-positive axons (red). The high-magnification insets (A–C) demonstrate that the neuroma contains both myelinated (MBP-positive, green) and nonmyelinated axons. In contrast, the healthy tibial nerve (D–F) comprises both myelinated and nonmyelinated axons (high-magnification insets) but retains a well-defined, organized fascicular structure. The images were acquired using a 20× objective and the high-magnification insets with 40× objective.

**Figure 3:**
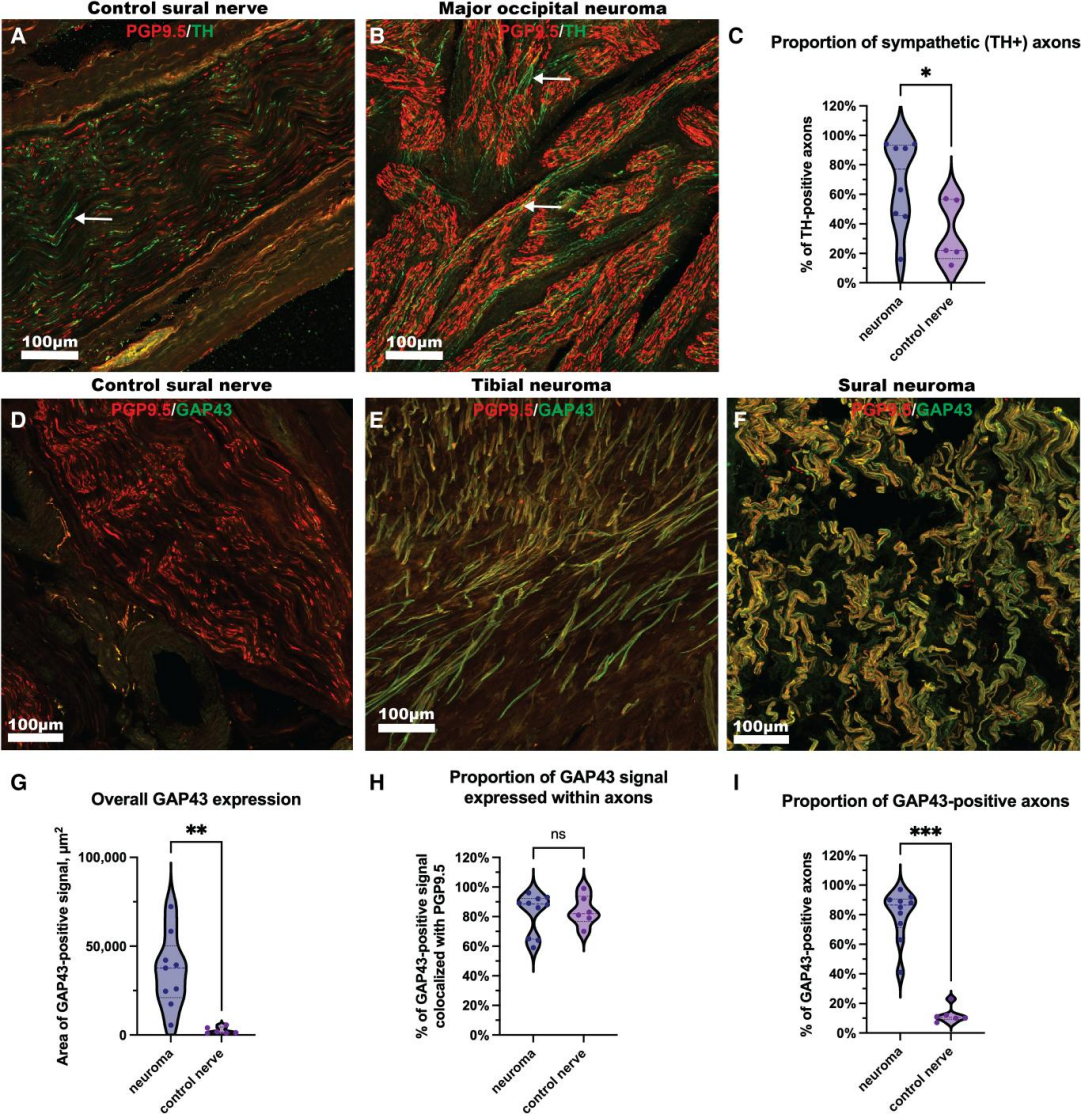
Painful neuromas contain a higher proportion of sympathetic nerve fibers (TH+) and exhibit increased regenerative potential (GAP43+) compared with healthy nerves. A) Representative image of a healthy sural nerve showing lower expression of TH+ axons (green, white arrows) compared with (B) a neuroma from the major occipital nerve. C) Quantification of TH-positive axons reveals a significantly higher proportion in painful neuromas (*n* = 8) compared with healthy nerves (*n* = 5; 67.3 ± 29.5% vs. 33.6 ± 21.3%, *P < 0.05, unpaired t test). D) The proportion of axons exhibiting regenerative potential (GAP43-positive, green) is lower in a healthy tibial nerve compared with neuromas from the (E) tibial and (F) sural nerves. G) Quantification of overall GAP43 expression shows significantly higher levels in painful neuromas (*n* = 10) compared with healthy nerves (*n* = 6) (35,934 ± 20,543 vs. 2,494 ± 1,902 µm^2^, **P < 0.01, unpaired t test). H) The majority of GAP43 expression is localized within axons in both groups, with no significant difference between painful neuromas and healthy nerves (88.5% [IQR 65–92] vs. 82% [IQR 77–94], *P* = 0.98, Mann–Whitney test). I) However, the proportion of GAP43-positive axons is significantly higher in painful neuromas (86.5% [IQR 71.3–90.5] vs. 10.5% [IQR 9.3–14.8], ***P < 0.0005, Mann–Whitney test). Scale bar: 100 µm.

The neural components of painful neuromas exhibit a disorganized axonal architecture (Fig. 2), characterized by a high regenerative capacity (growth-associated protein 43 [GAP43+]) and a notable prevalence of sympathetic nerve fibers (Fig. 3). All painful neuromas (*n* = 10), including neuromas-in-continuity (*n* = 4), lacked a fascicular structure compared with control nerves (Fig. 2A–F). Both myelinated (myelin basic protein [MBP]-positive signals targeting the Schwann cells and encircling the protein gene product 9.5 [PGP9.5] signal, green) and nonmyelinated axonal populations (only PGP9.5 signal [red]) were present in all painful neuromas (Fig. 2C).

The axonal populations within painful neuromas demonstrated a significantly higher proportion of tyrosine hydroxylase-positive (TH+) axons compared with control nerves (67.3 ± 29.5% vs. 33.6 ± 21.3%, *P* < 0.05), indicating an increased sympathetic contribution (Fig. 3A–C). While the role of increased sympathetic involvement in painful neuromas remains unclear, an increased abundance of blood vessels (α-SMA [alpha smooth muscle actin]-positive) was consistently observed in neuromas compared with control nerves (Fig. S1).

Overall, GAP43 expression was significantly elevated in painful neuromas compared with healthy nerves (35,934 ± 20,543 vs. 2,494 ± 1,902 µm^2^, *P* < 0.01; Fig. 3D–I). The majority of GAP43 expression was localized within axonal populations in both neuromas and healthy nerves (88.5%, interquartile range [IQR 65–92] vs. 82% [IQR 77–94], *P* = 0.98; Fig. 3H). Additionally, the proportion of axons within painful neuromas exhibiting detectable GAP43 expression was significantly higher compared with healthy nerves (86.5% [IQR 71.3–90.5] vs. 10.5% [IQR 9.3–14.8], *P* < 0.0005; Fig. 3I). These findings suggest that axons in painful neuromas retain their regenerative capacity.

### Painful neuromas exhibit increased calcitonin gene-related peptide expression compared with healthy nerves

**Figure 4:**
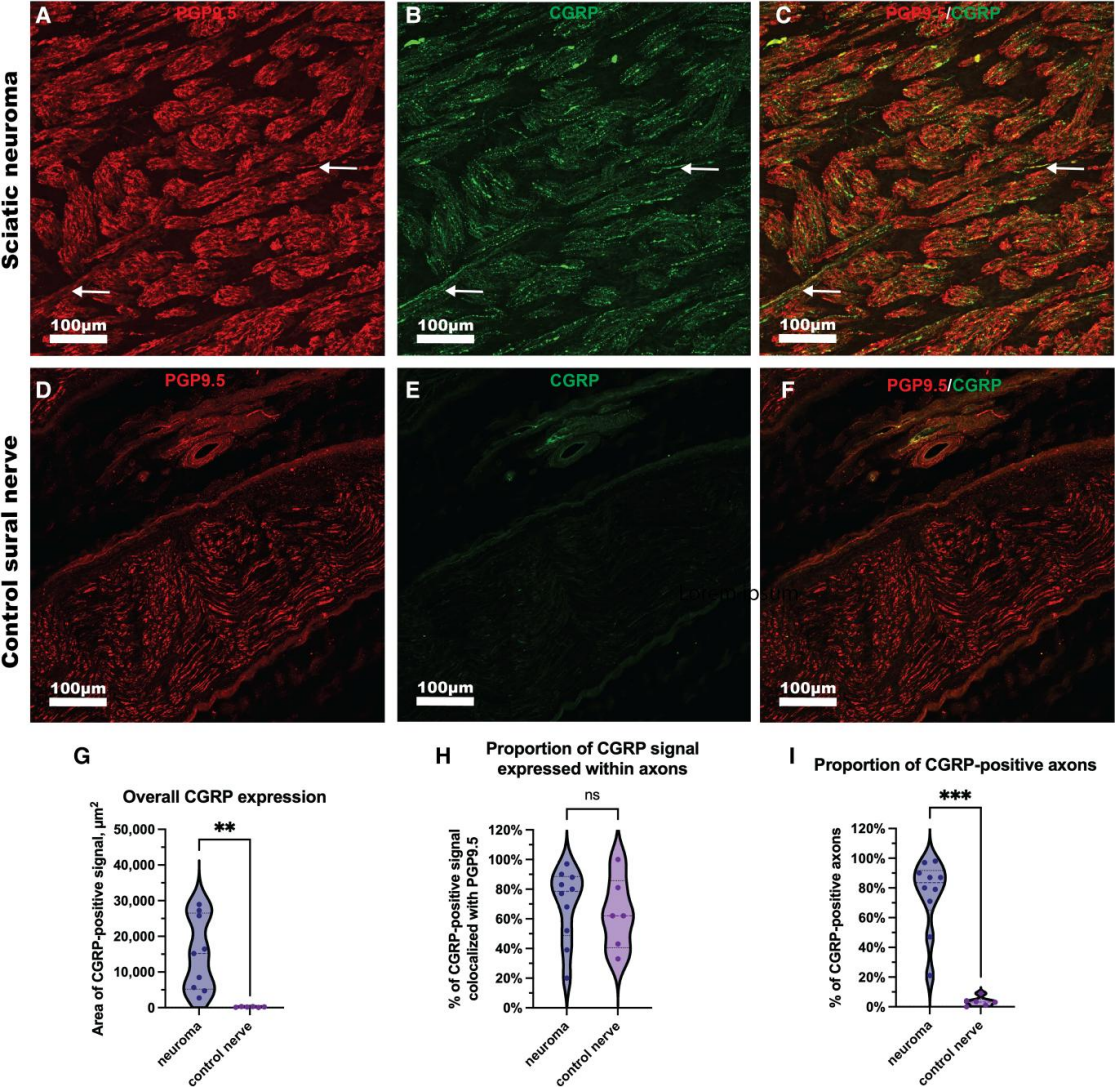
Painful neuromas show increased CGRP expression compared with healthy nerves. A–C) A sciatic neuroma exhibits a higher proportion of axons with elevated CGRP expression (green, white arrows) compared with (D–F) a healthy sural nerve. All images were acquired using a 20× objective with 1× optical zoom (effective magnification 20×). G) Overall CGRP expression is significantly higher in painful neuromas (*n* = 10) compared with healthy nerves (*n* = 6) (15,015 ± 10,294 µm^2^ vs. 198.8 ± 98.2 µm^2^, **P < 0.01, unpaired t test). H) The distribution of CGRP expression between axonal populations and the microenvironment does not differ significantly between neuromas and healthy nerves (69.4 ± 24.9% vs. 63.5 ± 24.5%, *P* = 0.65, unpaired t test). I) However, the proportion of CGRP-positive axons is significantly higher in painful neuromas (83.5% [IQR 65–91.8] vs. 3.2% [IQR 0.75–5.25], ***P < 0.0005, Mann–Whitney test). Scale bar: 100 µm.

The neural components of painful neuromas demonstrate an up-regulation of calcitonin gene-related peptide (CGRP; Fig. 4), a key nociceptive neuropeptide predominantly responsible for nociceptive perception (19, 20). The overall CGRP expression level, measured by the area covered with CGRP signal, was significantly higher in painful neuromas (15,015 ± 10,294 µm^2^) compared with healthy nerves (198.8 ± 98.2 µm^2^, *P* < 0.01; Fig. 4G). Additionally, the proportion of the total axonal population (PGP9.5-positive) exhibiting CGRP up-regulation was markedly increased in painful neuromas compared with healthy nerves (83.5% [IQR 65–91.8] vs. 3.2% [IQR 0.75–5.25], *P* < 0.001). However, the proportion of CGRP expression confined to axonal components versus the axon-free microenvironment (connective tissue, immune cells, and fibroblasts) did not significantly differ between neuromas and healthy nerves (69.4 ± 24.9% vs. 63.5 ± 24.5%, *P* = 0.65). These findings indicate that CGRP expression is up-regulated not only in the axonal populations of painful neuromas but also within the surrounding neuroma microenvironment (Movie S1). This suggests a potential phenotypic shift in injured axons toward a nociceptive profile, which may contribute to the pain-associated nature of neuromas.

### Painful neuromas exhibit altered expression of the mechanosensitive transmembrane channel Piezo2

**Figure 5:**
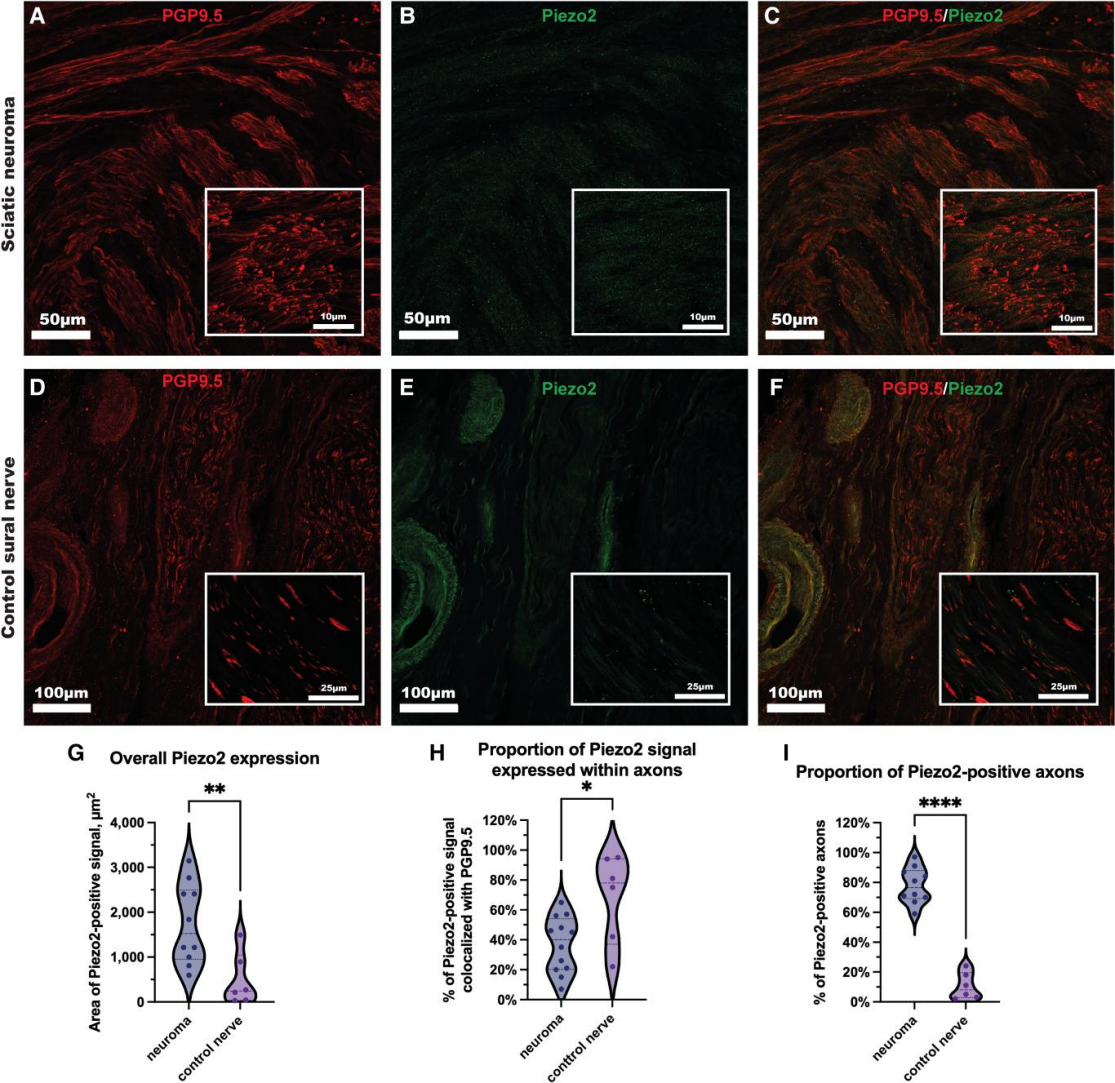
Painful neuromas exhibit increased expression of Piezo2 compared with healthy nerves. Representative images of sciatic neuroma (A–C) showing Piezo2 expression within the neuroma (high-magnification insets), compared with a healthy sural nerve (D–F). Images were acquired using a 20× objective with 1× optical zoom (effective magnification 20×), the high-magnification insets were acquired using a 40× objective with 1.5× optical zoom (effective magnification 60×). G) Overall Piezo2 expression area is significantly higher in painful neuromas (*n* = 10) compared with healthy nerves (*n* = 6; 1,739 ± 895 vs. 491 ± 583 µm^2^, **P < 0.01, unpaired t test). H) The proportion of Piezo2 expression localized to axonal populations is lower in painful neuromas compared with healthy nerves (36.8 ± 18.74% vs. 68.2 ± 29.7%, *P < 0.05, unpaired *t* test). I) The proportion of Piezo2-positive axons is significantly higher in painful neuromas (77.9 ± 12.0% vs. 10.5 ± 8.9%, ****P < 0.0001, unpaired t test).

While CGRP plays a key role in transsynaptic nociceptive transmission and neuroinflammatory responses, the transduction of mechanical forces and signals occurs via mechanosensitive transmembrane channels, such as the Piezo family (21). Our findings demonstrate significantly higher overall expression levels of Piezo2 in painful neuromas compared with healthy nerves (1,739 ± 895 vs. 491 ± 583 µm^2^, *P* < 0.001; Fig. 5). Additionally, the proportion of axonal components exhibiting Piezo2 up-regulation was significantly higher in painful neuromas compared with healthy nerves (77.9 ± 12.0% vs. 10.5 ± 8.9%, *P* < 0.0001), suggesting that axonal populations in painful neuromas are more susceptible to mechanical stimulation. Interestingly, the proportion of Piezo2 expression confined to axonal populations was lower in painful neuromas than in the axonal populations of healthy control nerves (36.8 ± 18.74% vs. 68.2 ± 29.7%, *P* < 0.05; Fig. 5H). This finding suggests that Piezo2 up-regulation extends beyond neural populations in painful neuromas, occurring within the broader neuroma microenvironment, including non-neuronal cellular components.

### High expression of low-threshold sodium channel Nav1.3 suggests increased hyperexcitability in painful neuromas

**Figure 6:**
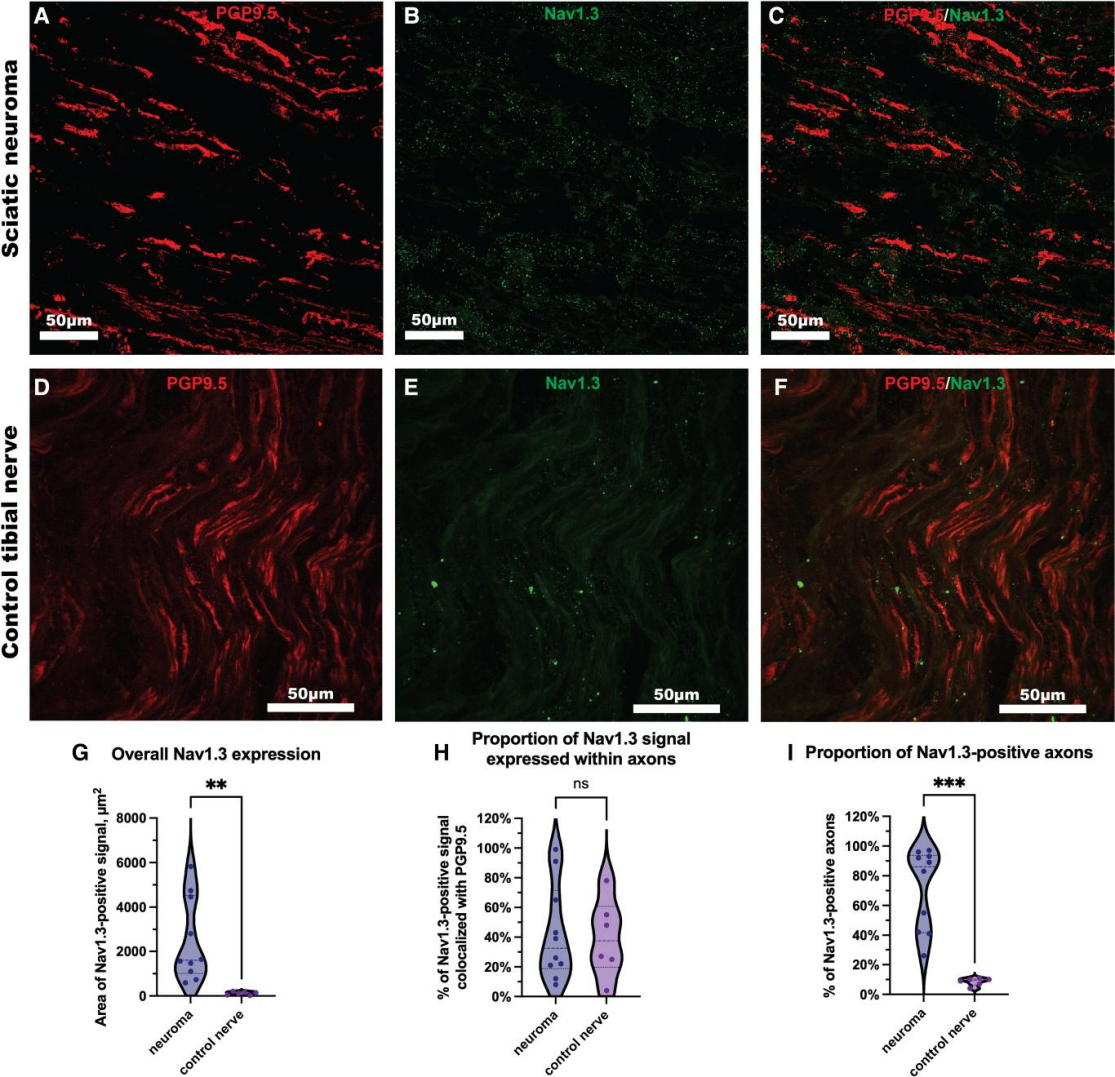
Painful neuromas exhibit hyperexcitable characteristics due to increased Nav1.3 expression. A–C) Representative high-magnification images of sciatic neuroma showing elevated Nav1.3 expression (green) compared with (D–F) a healthy tibial nerve. Images were acquired using a 40× objective with 1.5× optical zoom (effective magnification 60×). G) Overall Nav1.3 expression area is significantly higher in painful neuromas (*n* = 10) compared with healthy nerves (*n* = 6; 2,493 ± 1,867 vs. 116 ± 75.4 µm^2^, *P* < 0.01, unpaired t test). H) The distribution of Nav1.3 expression between axonal populations and the microenvironment does not differ significantly between neuromas and healthy nerves (42.6 ± 32.2% vs. 39.5 ± 26.1%, *P* = 0.84, unpaired t test). I) The proportion of Nav1.3-positive axons is significantly higher in painful neuromas (86% [IQR 42–94] vs. 9.5% [IQR 6.25–10.25], *P* < 0.0001, Mann–Whitney test). Scale bar: 50 µm.

While ionotropic sodium transmembrane channels respond to various stimuli and propagate signals upstream via axons, different channels exhibit varying activation thresholds. Our findings indicate that the low-threshold sodium channel Nav1.3 is up-regulated in painful neuromas (Fig. 6A–C). The overall expression of Nav1.3 was significantly higher in painful neuromas compared with healthy nerves (2,493 ± 1,867 vs. 116 ± 75.4 µm^2^, *P* < 0.01). Additionally, the proportion of axonal components (PGP9.5-positive) with high Nav1.3 expression was significantly greater in painful neuromas compared with axons in healthy nerves (86% [IQR 42–94] vs. 9.5% [IQR 6.25–10.25], *P* < 0.0001). However, the proportion of Nav1.3 expression confined to axonal components versus the axon-free microenvironment (connective tissue, immune cells, and fibroblasts) did not differ between neuromas and healthy nerves (42.6 ± 32.2% vs. 39.5 ± 26.1%, *P* = 0.84). Overall, the up-regulation of the low-threshold sodium channel Nav1.3 in painful neuromas suggests increased susceptibility to excitation in response to various stimuli.

## Discussion

Neuropathic pain affects a significant portion of the population, with painful neuromas representing a well-defined yet poorly understood etiology. Despite advancements in surgical approaches, the outcomes may vary and the precise mechanisms underlying neuroma formation and associated pain remain elusive (6, 9, 22). In this study, we demonstrated distinct alterations in nociceptor marker expression within painful neuromas, highlighting key molecular changes that may contribute to their pathophysiology. Our findings indicate a phenotypic shift in axonal components within painful neuromas compared with healthy nerves, characterized by “increased CGRP expression.” This shift suggests a transition toward a nociceptive profile, likely contributing to increased pain perception. Furthermore, we observed an “up-regulation of the mechanosensitive Piezo2 channel” and the “low-threshold sodium channel Nav1.3,” underscoring the mechanosensitive and hyperexcitable nature of painful neuromas. These findings not only provide insight into the neural molecular architecture of painful neuromas but also suggest potential targets for novel therapeutic interventions.

Pain perception is an evolutionary adaptation that serves as a critical warning system (23, 24). Even animals lacking neurons can perceive pain through molecular mechanisms, relying on intracellular molecules such as glutamate, ATP, and peptides to transduce pain signals (25, 26). This highlights the ancient biological foundation of pain perception. However, in the context of nerve injury, this mechanism can become maladaptive, leading to chronic neuropathic pain. The peripheral nervous system exhibits a remarkable regenerative capacity, allowing injured axons to regrow after trauma or injury (27). While neurons in the central nervous system show limited regenerative potential, glial cells in both the central and peripheral nervous systems can proliferate and support repair (28). Axonal regeneration, an ancient evolutionary phenomenon, has been documented across various species, including lower invertebrates (29). In particular, *Caenorhabditis elegans* axons exhibit extensive regenerative properties, akin to those observed in humans (30, 31), and similar regenerative capabilities are found in other species, such as zebrafish and amphibians, further supporting the long-standing evolutionary presence of axonal repair mechanisms (32). However, while axons have been capable of regrowth for millions of years, neuroma formation appears to be a relatively recent evolutionary development, with the earliest documented descriptions emerging from ornithological studies (33).

This shift raises significant questions about the evolutionary significance and biological role of neuroma formation, particularly how and why this maladaptive process emerged despite the long-standing ability of axons to regenerate. Our findings demonstrate that painful neuromas exhibit high levels of GAP43 expression, indicating an ongoing regenerative state. This highlights the preserved regenerative potential of axonal components alongside the up-regulation of other nociceptor markers. Moreover, previous studies suggest that injured axons become hyperexcitable shortly after injury, resulting in increased mechanical sensitivity (34, 35). The link between ongoing axonal growth and up-regulated nociceptor markers raises a critical question: Does the regenerative capacity of injured axons contribute to increased pain sensitivity? If so, could inhibiting axonal regeneration mitigate neuroma-associated pain? Targeting GAP43 expression in painful neuromas could represent a promising therapeutic approach, although further studies are necessary to evaluate its clinical potential (36, 37).

Deininger et al. recently characterized the Schwann cell repair phenotype and inflammatory changes in a larger cohort of human neuromas (38). Other prior studies document Nav1.7 and Nav1.8 up-regulation in painful neuromas (39, 40). In contrast, our study focuses on the axonal nociceptive and mechanosensitive molecular signature (CGRP, Piezo2, and Nav1.3) that directly underlies pain generation and hyperexcitability. While axonal components are the primary mediators of neuroma-associated pain, the interactions between regenerating axons and the neuroma microenvironment remain poorly understood. Specifically, the role of immune cell infiltration and transcriptomic remodeling in pain generation warrants further investigation (9, 20). Our data show that CGRP expression is expressed in 84% of axonal components within painful neuromas compared with 3% in control nerves, suggesting a functional shift toward nociceptive signaling or an increase in the fraction of peptidergic axons in neuromas compared with control nerves. While the findings are predominantly only descriptive and not mechanistic in nature, this phenotypic switch may contribute to the increased pain sensitivity observed in neuroma patients. Although C-fibers and other nociceptive axons are responsible for pain perception in healthy state, differences in molecular expression patterns across sensory and mixed nerves remain unclear due to limited sample sizes of this study.

In addition to increased CGRP expression in neuromas compared with control nerves, our study highlights a distinct pattern of nociceptive reprogramming within the axolemma, characterized by the up-regulation of Piezo2 and Nav1.3 channels. Clinically, touch-evoked pain is a hallmark of neuroma-associated neuropathy, and the increased proportion of Piezo2-expressing axons (78% vs. 11%) aligns with this observation (21). Recent studies show the association of Piezo2 not only with mechanosensitive transduction but also with transduction of pain (41, 42). Another highly recognizable feature of painful neuromas is their hyperexcitability. Biologically similar to the neurophysiological behavior of cortical neurons in epilepsy patients, painful neuromas exhibit hyperexcitability and subsequent spontaneous action potentials (43, 44). The up-regulation of the voltage-gated sodium channel Nav1.3 in dorsal root ganglion neurons following peripheral axonal injury is well established (45, 46). Nav1.3 exhibits rapid repriming kinetics and distinctive inactivation properties that render it particularly suited to generating neuronal hyperexcitability (47). In some clinical cases of epilepsy, it has been found that a low-threshold sodium channel, Nav1.3, is up-regulated in these neurons (48, 49). Interestingly, we found the same up-regulation of Nav1.3 in the axonal components of painful neuromas (86% vs. 9.5%). This suggests that axonal populations within painful neuromas undergo transcriptomic reprogramming, switching their excitability patterns toward a hyperexcitable state, similar to what is observed in epilepsy. These parallels suggest that transcriptomic reprogramming in axons of painful neuromas may contribute to the hyperexcitable nature of neuromas.

The up-regulation of nociceptive ion channels in neuromas presents a promising opportunity for focal pain management strategies. Unlike systemic neuropathic pain syndromes, neuromas are localized lesions, allowing for precise therapeutic targeting (50, 51). Pharmacological agents targeting transmembrane channels, such as Piezo2 and Nav1.3 inhibitors, may offer an effective strategy for alleviating neuroma-associated pain while minimizing systemic side effects (48). Additionally, CGRP-targeting therapies, which have shown efficacy in migraine treatment, could be explored for neuroma pain relief (52). Current clinical trials are investigating alternative sodium channel targets (e.g. Nav1.7, Nav1.8), which have been implicated in various pain conditions, including diabetic neuropathy, inflammatory pain, and postsurgical pain (50, 53–56). These promising studies show potential for developing targeted treatment options for painful neuromas.

One of the key unresolved questions is whether molecular expression patterns differ across upper vs. lower extremity nerves, sensory vs. mixed nerves, or patient demographics. Some neuroma-to-neuroma variation in the expression of the investigated markers was observed. However, given the limited sample size, the study is not powered to test for meaningful associations between individual expression profiles and specific pain phenotypes, and any such relationships must be regarded as descriptive only. While all patients demonstrated positive Hoffmann–Tinel sign (57), the heterogeneity in pain descriptors across our cohort (burning [*n* = 7/10], sharp [*n* = 8/10], and paresthesia [*n* = 6/10]) reflects the complex nature of neuroma pain. Raasveld et al. have shown that atypical neuromas with disrupted fascicular architecture are associated with the highest pain scores, whereas preserved fascicles are protective (58).

The major limitation of this study is the modest sample size and the exploratory nature of our findings. While our data reveal notable molecular differences between painful neuromas and healthy nerves, the lack of a regenerating-nerve control group means we cannot definitively distinguish between markers specific to pathological neuroma formation and those associated with general physiological nerve regeneration. Furthermore, our use of healthy tibial and sural nerves as a baseline—while necessary due to the practical challenges of human tissue acquisition—introduces potential anatomical variability. Consequently, our findings are best interpreted as identifying candidate molecular alterations that warrant further investigation in larger, controlled cohorts. Due to the limited sample size (*n* = 10) in this study, we were unable to perform subgroup analyses. However, the consistent up-regulation of CGRP, Piezo2, and Nav1.3 across all studied neuromas suggests a reproducible trend. To further elucidate the molecular landscape of painful neuromas, we are in the process of establishing a comprehensive biobank with larger patient cohorts. Future research will incorporate single-cell RNA sequencing, spatial transcriptomics, and proteomics to provide a detailed understanding of neuroma biology. Additionally, advanced tissue-clearing and three-dimensional imaging techniques will overcome the limitations of traditional confocal microscopy, enabling more precise visualization of neuroma architecture. Future studies focusing on immune–axon interactions, transcriptomic alterations, and targeted therapeutics will be essential for improving clinical management and alleviating neuropathic pain in affected patients.

## Materials and methods

### Human subjects' enrollment

Sixteen subjects were recruited for this study regardless of their sex/gender, or ethnicity at the Massachusetts General Hospital from January 2023 to November 2024. In total, four patients were excluded from the study due to poor specimen conditions for immunofluorescent analysis. Nine patients (with 10 neuromas in total) were included in the study, who underwent neuroma excision and three patients (with total 6 healthy nerves) were included who underwent lower extremity amputations. Following inclusion criteria were used in the study: undergoing TMR and/or RPNI surgery, >18 years, competent adults able to consent on their own behalf for surgery, literate in English (for consenting purposes). Exclusion criteria were pregnant women, unable to give informed consent. The presence of painful neuromas was diagnosed by a fully trained attending in plastic surgery according to our diagnostic criteria (59). Following neuroma excision, the proximal stump of the nerve was treated with active nerve management. Patient-specific demographic data included sex, date of birth, age at the first surgical procedure, location of neuromas and pain, time following neuroma formation (nerve injury), and disease-associated comorbidities (Table 1). Informed consent was obtained from all patients, and ethical commission approval was given by the Massachusetts General Brigham Institutional Review Board (protocol numbers: 2020P004060 and 2020P003555).

**Table 1:** Baseline characteristics.

	Neuroma group (*n* = 9 patients with *n* = 10 neuromas)	Healthy nerve group (*n* = 3 patients with *n* = 6 nerves)
Sex		
Female, *n* (%)	5 (56)	1 (33)
Male, *n* (%)	4 (44)	2 (67)
Age at surgery, years, mean ± SD	43 ± 18	56 ± 16
Nerves, *n* (%)	10 (100)	6 (100)
Major occipital nerve	2 (20)	n/a
Ulnar nerve	1 (10)	n/a
LABC	1 (10)	n/a
Sciatic nerve	2 (20)	n/a
Tibial nerve	1 (10)	3 (50)
Sural nerve	2 (20)	3 (50)
Neuroma in continuity, *n* (%)	4 (40)	n/a
Amputation, *n* (%)	4 (40)	6 (100)
Time from initial injury to specimen harvesting (surgery), months, median (IQR)	10 (5.3–24)	n/a
Pain score at surgery, mean ± SD	7.4 ± 1.2	n/a

LABC, lateral antebrachial cutaneous nerve; n/a, not applicable.

### Collection and specimen processing

The neuroma specimens were harvested intraoperatively following surgical excision while the healthy nerves were dissected from the amputated extremities following amputation. Harvested samples were immediately fixated by immersion in 4% paraformaldehyde diluted in 0.1 M phosphate-buffered saline (PBS), pH 7.4, at +4 °C, for 12–24 h. Afterward, the samples were extensively washed with PBS, pH 7.4, followed by dehydration in increasing sucrose/PBS solutions (10, 25, and 40%) for 24 h in each at +4 °C. Afterward, the samples were embedded in Tissue-Tek O.C.T. tm Compound (Thermo Fisher Scientific, Agawam, MA, United States) and stored at −80 °C. Nerves were cut into 10–50 μm thick cross-sections using a cryostat (Thermo Scientific Microm HM 550 MVPD, Cryostat 956544D, Cridersville, OH, United States). In larger neuroma specimens, unequal tissue fixation penetration between central and peripheral regions may contribute to apparent differences in staining intensity and should be considered when interpreting qualitative image comparisons.

### Immunofluorescence staining

Following cryosectioning, the specimens underwent immunofluorescence staining using antibodies against specific molecular markers. The double immunofluorescent staining was used, as previously established (60, 61). Primary antibodies were obtained from Merck/Millipore (Temecula, CA, United States), Cell Signaling Technologies (Danvers, MA, United States), Bio-Rad (Hercules, CA, United States), Alomone Labs (Jerusalem, Israel), ProteinTech (Rosemont, IL, United States), and Abcam (Cambridge, United Kingdom). Antibodies from Merck/Millipore included rabbit anti-TH (AB152, lot number: 4003833), anti-Piezo2 (HPA031975; lot number: 000015850), rabbit anti-GAP43 (AB5312; lot number: 3915631), and polyclonal rat anti-MBP (catalogue number MAB386; lot number: 4001919). From Cell Signaling Technologies, rabbit anti-CGRP (catalogue number D5R8F; lot number: 2798662) was obtained. From Bio-Rad, PGP9.5 (catalogue number 7863-1004; lot number: 158905) was obtained. Antibodies from Agilent Technologies, Alomone Labs (rabbit anti-Nav1.3; ASC-004; lot number: ASC004AN1202), ProteinTech (rabbit anti-SMA-α; AB5694; lot number: 1038192-1), and Abcam (anti-ColIII; 22734-1-AP; lot number: 00125919) were included. Anti-PGP9.5 was used at a concentration of 1:500, anti-MBP at 1:200, anti-TH at 1:250, anti-GAP43 at 1:500, anti-CGRP at 1:500, anti-Piezo2 at 1:100, anti-Nav1.3 at 1:100, anti-SMA-α at 1:500, and anti-ColIII at 1:500. Secondary antibodies conjugated with alexa fluor 488 or 568 were obtained from Thermo Fisher (Waltham, MA, United States). All secondary antibodies were used at the concentration of 1:500. Anti-PGP9.5 marker labels a broader range of axons, including myelinated and unmyelinated fibers, as well as nerve endings. It provides a more comprehensive view of the nerve structure, including both sensory and autonomic fibers. Nociceptive afferent fibers were visualized by anti-CGRP and sympathetic nerve fibers by anti-TH. The myelinated nerve fibers were visualized with anti-MBP, which stains myelin sheaths. Piezo2 is a mechanosensitive transmembrane protein responsible for sensing mechanical stimului. Nav1.3 is a low-threshold transmembrane sodium channel.

Sections of the neuroma and nerve samples were stained with (i) anti-PGP9.5 and anti-CGRP, (ii) anti-PGP9.5 and anti-MBP, (iii) anti-PGP9.5 and anti-TH, (iv) anti-PGP9.5 and anti-Piezo2, (v) anti-PGP9.5 and Nav1.3, (vi) anti-PGP9.5 and ColIII, and (vii) anti-PGP9.5 and α-SMA. Before labeling, frozen sections were air dried followed by incubation in 10% normal goat or rabbit serum (Invitrogen, Carlsbad, CA, United States) in PBS containing 0.1% Triton or 0.5% for sections over 50 µm. Thereafter, sections were incubated for 48 h with the primary antibodies, washed with PBS, and incubated for 2 h with the secondary antibodies. Finally, the tissue was rinsed again in PBS and mounted in fluorescence mounting medium (Technologies, Santa Clara, CA, United States). To minimize batch effects, neuroma specimens and their corresponding healthy nerve controls were always processed in the same immunostaining run.

### Confocal imaging

Fluorescently labeled neuroma and control nerve samples were imaged on a confocal laser scanning microscope (Nikon ECLIPSE Ti2 with DarkPanel Cage Incubator, Oko Labs). For each specimen, z-stacks of optical sections (0.5–1 µm step size) were acquired through regions of interest at a resolution of 1,024 × 1,024 pixels. Double-label images were obtained using 488 and 568 nm excitation lines, and three-dimensional projections were generated using the manufacturer's confocal software. To ensure quantitative rather than purely qualitative analysis, we acquired ten longitudinal sections spanning ∼50 µm tissue depth per specimen and sampled at least three nonoverlapping regions of interest per section. Quantitative measurements for each marker were averaged across all sections and regions for a given specimen.

Quantitative images were acquired using fixed confocal settings within each staining panel. For Alexa Fluor 488–conjugated secondaries (CGRP, TH, and GAP43), the 488 nm laser was set to 2% power, with detector high-voltage (HV) gain between 100 and 120 and digital offset fixed at 0; these parameters were kept identical for all neuroma and control samples stained for these markers. For Piezo2 and Nav1.3 (also detected with AF-488), the 488 nm laser power was increased to 5%, again with HV gain in the 100–120 range and offset at 0, and these settings were held constant across all specimens in this panel. For PGP9.5 (568 nm channel), the 568 nm laser was operated at 5% power with HV gain 100–120 and offset 0, identical for all samples in that panel. Within each staining panel, pinhole size, pixel size, and sampling speed were likewise fixed for all neuroma and healthy nerve sections. No automatic exposure, auto-gain, or postacquisition contrast/gamma adjustments were applied to images used for quantitative analysis.

The intensities and cross-sectional areas of fluorescent signals were quantified using QuPath (version 0.5.1) (62). For quantitative colocalization analysis, cross-sections stained with anti-PGP9.5 and AF-488–conjugated secondaries (CGRP, TH, GAP43, Piezo2, and Nav1.3) were analyzed. PGP9.5-positive axons were segmented using the pixel classification module, which combines intensity and texture features and is recommended over simple global thresholding for heterogeneous tissue. The cell detection module was then applied to identify AF-488–positive signals for each marker.

Thresholds and classifier parameters were determined in a two-step, batch-wise fashion. First, for each staining panel, a pixel classifier was trained on a representative subset of neuroma and control sections acquired with identical confocal settings, using manually annotated examples of positive and negative signal. Once optimized, these thresholds and classifier settings were applied unchanged to all images within the same staining batch; thresholds were not tuned on a per-image basis. This approach follows recommended practice for applying consistent intensity thresholds in quantitative confocal microscopy and digital pathology workflows.

For each image, the cross-sectional areas of PGP9.5-positive and AF-488–positive regions were measured, and colocalization quotients were calculated from the overlap between both signals. AF-488 signal intensity was quantified separately within PGP9.5-positive axonal regions and in PGP9.5-negative areas. All automated segmentations and classifications were visually inspected, and occasional obvious misclassifications were corrected manually to ensure accurate identification of PGP9.5-positive axons and AF-488–positive signals.

### Statistical analysis

Data were analyzed using GraphPad Prism 10 (GraphPad Software, San Diego, CA, United States). Parametric values are expressed as mean with SD, and nonparametric values are expressed as median with IQR. To check the normal distribution of the variables, the Kolmogorov–Smirnov test was used. Descriptive statistics was used for baseline characteristics of the enrolled patients. Parametric data were compared between the painful neuromas and healthy nerves using a unpaired t test, while nonparametric data were compared between the groups using a Mann–Whitney *U* test. Results with a *P*-value of <0.05 were considered significant.

## Supplementary Material

pgag257_Supplementary_Data

## Data Availability

Data used in the study are included in the manuscript and supporting information. Partial restrictions to the data or physical materials apply: patient-derived raw data are not publicly available due to privacy and ethical restrictions, but de-identified datasets may be available from the corresponding author on reasonable request, pending institutional approval.
