# Supplementary material for: Painful neuromas exhibit axonal phenotypic shift via nociceptive and mechanosensitive marker up-regulation
Source: PNAS Nexus. 2026 Aug 11;5(8):pgag257. doi: 10.1093/pnasnexus/pgag257 (PMC13459132; doi:10.1093/pnasnexus/pgag257)
Supplement: Supplementary file 1 [file pgag257_supplementary_data.zip › PNASNEXUS-PNASNEXUS-2025-01219RRR-s01.docx]

Supplementary Materials for

**Painful neuromas exhibit axonal phenotypic shift via nociceptive and mechanosensitive marker upregulation**

Vlad Tereshenko^1^, Floris V. Raasveld^1^, Charles D. Hwang^1^, Madison R. Hussey^1^, Benjamin Johnston^2^, Ian L. Valerio^1^, William G. Austen Jr.^1^, Brian J Wainger^3^, William Renthal^4^, Kyle R. Eberlin^1^*

Corresponding author: [keberlin@mgh.harvard.edu](mailto:keberlin@mgh.harvard.edu)

**The PDF file includes:**

Fig. S1

**Other Supplementary Materials for this manuscript include the following:**

Movie S1

**Fig S1. Neural components with vascular structures in painful neuromas and healthy nerves.** The painful sural neuroma (**A-C**) contains abundance of vascular structures (α-SMA-positive, green) compared to healthy tibial nerve (**D-F**).

**Movie S1. Three-dimensional reconstruction of sural neuroma stained with PGP9.5 (red) and CGRP (green).**
